# Steroidal Saponins with Plant Growth Stimulation Effects; *Yucca schidigera* as a Commercial Source

**DOI:** 10.3390/plants11233378

**Published:** 2022-12-05

**Authors:** Alexandra G. Durán, Juan M. Calle, Davinia Butrón, Andy J. Pérez, Francisco A. Macías, Ana M. Simonet

**Affiliations:** 1 Allelopathy Group, Department of Organic Chemistry, Campus de Excelencia Internacional (ceiA3), Institute of Biomolecules (INBIO), School of Science, University of Cádiz, C/República Saharaui 7, 11510 Cádiz, Spain; 2 Departamento de Análisis Instrumental, Facultad de Farmacia, Universidad de Concepción, Concepción 4070386, Chile

**Keywords:** *Agave*, *Yucca schidigera*, steroidal saponin, plant growth stimulation, hormesis, wheat coleoptile bioassay, agameroside I

## Abstract

Plant growth-stimulation bioactivity of triterpenoid saponins is well known, especially for oleanane-type compounds. Nevertheless, a few phytotoxicity bioassays performed on some steroidal saponins have shown hormesis profiles and growth stimulation on *Lactuca sativa* roots. The focus of the work described here was on the use of the wheat coleoptile bioassay to evaluate plant growth stimulation, and on the search for a commercially available source of active saponins by bio-guided fractionation strategy. Selected saponins were tested and a cluster analysis showed that those saponins with a sugar chain of more than five units had a hormesis profile, while saponins with growth enhancement had fewer sugar residues. Two saponins showed similar activity to the positive control, namely the phytohormone indole-3-butyric acid (IBA). As a potential source of these metabolites, a commercial extract of *Yucca schidigera* used as a fertilizer was selected. Bio-guided fractionation led to the identification of two fractions of defined composition and these showed stimulation values similar to the positive control. It was observed that the presence of a carbonyl group at C-12 on the aglycone skeleton led to improved activity. A saponin-rich fraction from *Y. schidigera* could be proposed to enhance crop quality and production.

## 1. Introduction

The role of saponins in plant growth stimulation has been well known since the mid-twentieth century. It has been demonstrated that the treatment of tomato seeds and cereals with saponins at low concentrations promotes both the germination and the growth rate [1]. This observation was explained by the surface-active properties and water absorption modulation of these compounds [2].

The results of some studies suggest that saponins in aqueous solution form aggregates with a molecular weight of 10^6^ Da [3]. In respect of this approach, a mechanism of action for saponins has been proposed. Saponin molecules could form a layer that surrounds the roots, and this will transport water to the roots. The saponins would act as a hydrophilic bilayer that would favor the development of the plant and these would preclude the uptake of water and nutrients. For the seed that is germinating, it is advantageous to be surrounded by an appropriate layer of saponins, with this being the possible ecological role of these compounds [4].

In particular, the growth-enhancing effects of triterpenoid saponins have been described in previous publications, especially for the oleanane-type saponins from tea seeds. A plant growth regulator has been patented [5] and this contains metabolites that produce stimulation at low concentrations (0.1–5 ppm) and inhibition at higher concentrations (50–1000 ppm) in different plant species, including lettuce, tomato, pumpkin, and leek. Furthermore, these saponins are considered as natural surfactants that are beneficial for soil remediation [6].

The stimulatory effects of the crude extract of triterpenoid saponins from *Vigna radiata* L. (mungbean or green gram) have been evaluated on the same plant. It is worth highlighting the growth-enhancing effects observed for this plant (up to 25% weight increase) after 84 days of germination with concentrations in the range 150–450 ppm of the crude extract [7]. The component from this crude extract that has been studied in greater depth is the oleanane-type saponin chromosaponin I. This compound showed stimulatory effects on lettuce roots (*Lactuca sativa* L.) and gave a growth of 90% with respect to the control after 42 h of treatment and a concentration of 3 mM. The stimulation was detected 6 h after treatment with chromosaponin I and the accelerated growth continued for a further 17 h, thus confirming the stimulating effect on the roots by this saponin [8].

**Figure 1:**
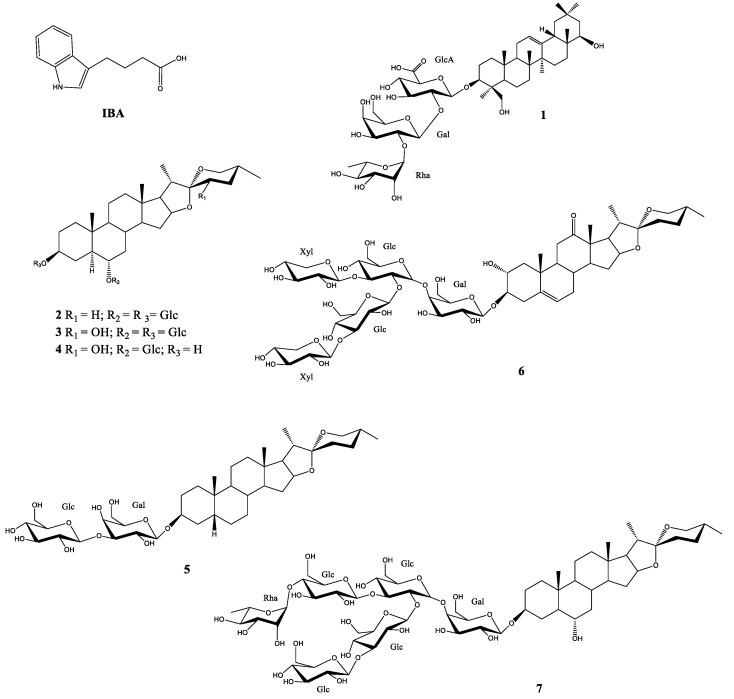
Saponins tested on etiolated wheat coleoptile elongation with IBA as positive control.

In contrast to the above, soyasaponin I (**1**) (Figure 1), which is structurally derived from chromosaponin I, displayed a milder growth-promoting effect [9].

The effect of chromosaponin I on the roots of *Arabidopsis* has also been studied [10] and it was noted that the growth stimulation observed is due to cell elongation and cell proliferation. It is believed that this saponin produces this effect by interfering with the plant hormone ethylene [11]. It was also found that this saponin specifically interacts with the AUX1 protein in regulating the gravitropic response of *Arabidopsis* roots [12]. In all of these studies, it was found that the mode of action is not merely due to a surface-active effect, as initially proposed.

Other investigations have focused on the growth enhancement of oleanane-type saponins on roots and especially on shoots of *Lolium perenne* (ryegrass), with stimulation percentages higher than 20% obtained at a concentration of 0.2 mg/mL. Slight stimulation of some of the saponins tested on the roots of *Solanum lycopersicum* (tomato) and *L. sativa* has also been observed [13].

Regarding steroidal saponins, these compounds have generally shown phytotoxic effects [14,15,16,17], although in some cases growth stimulation (**4**, 18% at 10 μM) and hormesis (**6**, 16% at 10 μM) [18,19] have been observed when these metabolites were tested on the roots of *L. sativa*.

In order to gain further insights into the potential of steroidal saponins, it was proposed to employ a specific bioassay to evaluate the growth stimulating effects, since the phytotoxicity bioassay is optimized to measure inhibitory effects. On applying the appropriate bioassay, a bio-guided search for commercially available sources of steroidal saponins with stimulatory potential was also proposed. 

## 2. Results

### 2.1. Stimulation Activity of Steroidal Saponins

The etiolated wheat coleoptile bioassay (*Triticum aestivum* L.) was used to evaluate the potential of steroidal saponins as growth promoters [20,21]. In this bioassay, the parameter evaluated is the cell growth as an increase in the length of the coleoptile. Activity is measured as the percentage variation of coleoptile growth after treatment of the samples with respect to control (buffered aqueous solution without the compound to be evaluated). Positive activity values indicate a growth greater than that observed for the control solution, while negative values indicate growth inhibition.

**Figure 2:**
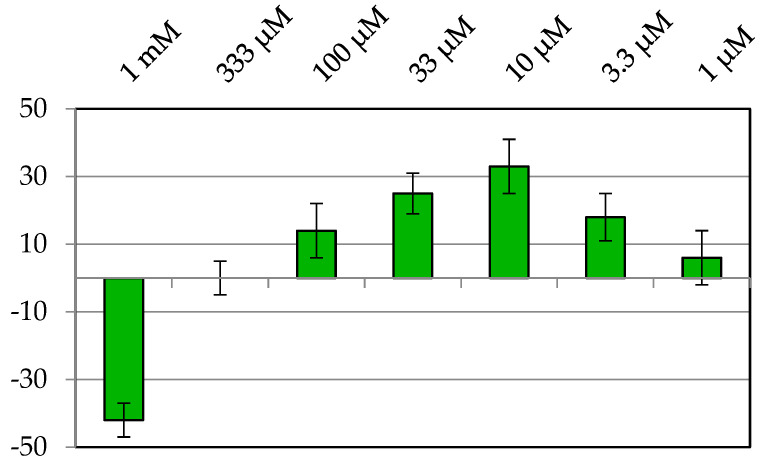
Effect of the phytohormone IBA on etiolated wheat coleoptiles. Experiments were performed in triplicate and values are expressed as percentage difference from control and as mean ± SD. Coleoptile elongation was measured where negative values indicate growth inhibition and positive values represent growth stimulation.

Firstly, the phytohormone indole-3-butyric acid (IBA) [22] was tested in this bioassay due to its structural similarity to the wheat auxin (IAA), its ready availability, and its affordable price. IBA was tested at concentrations from 1 mM to 10 nM, and it was observed that from 1 µM (Figure 2) the stimulatory activity was not kept. A dose-dependent response profile can be observed and values higher than 30% growth enhancement were achieved at 10 µM. In contrast, an inhibitory effect was produced at the highest concentration. 

**Figure 3:**
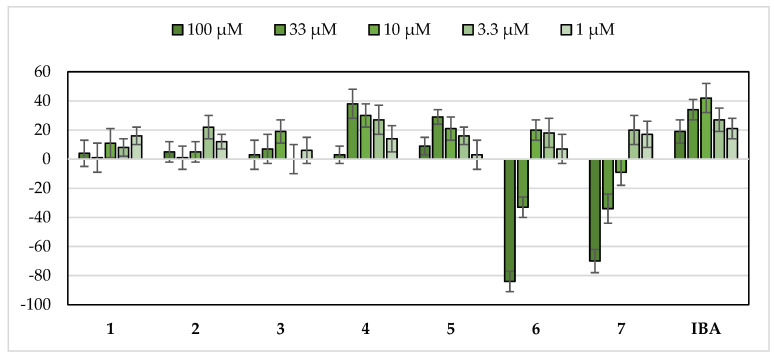
Effects of the selected steroidal saponins (**2**–**7**) on etiolated wheat coleoptile elongation. Values are expressed as percentage difference from control and as mean ± SD. Experiments were performed in triplicate where negative values indicate growth inhibition and positive values represent growth stimulation.

**Figure 4:**
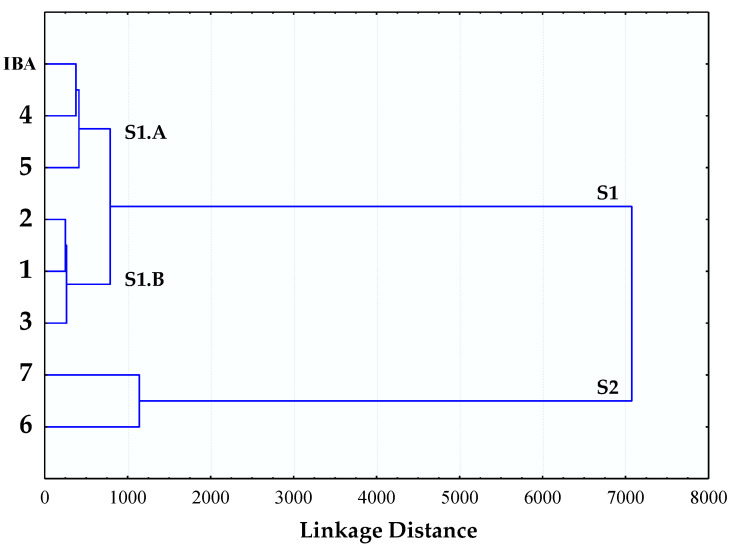
Cluster analysis of the selected steroidal saponins on etiolated wheat coleoptile elongation.

Once it had been confirmed that this bioassay was able to measure the stimulatory activity, a selection of saponins (**2**–**7**) available in our laboratory were chosen for testing (Figure 1). The saponins were evaluated in the etiolated wheat coleoptile bioassay between 100 and 1 µM (Figure 3), with IBA employed as positive control and the triterpenoid saponin soyasaponin I (**1**) included due to its bibliographic background of plant growth stimulation and its availability in our laboratory. A cluster analysis was performed to classify the test compounds according to their stimulatory activity and properties (Figure 4).

Two groups can be clearly distinguished in the cluster analysis (S1 and S2). Saponins with a sugar chain of more than five units (**6** and **7**) are found in the S2 group, which showed a strong inhibitory activity at the first two concentrations (100 and 33 µM) and a moderate activity in the range from 10 to 1 µM.

In contrast to the above, the saponins characterized by a short sugar chain that did not show inhibitory activity at the concentrations tested are grouped in the S1 cluster. Additionally, two subgroups can be differentiated within this latter group, namely S1.A and S1.B. The first subgroup is formed by compounds **4** and **5**, which have a good stimulatory activity profile, and the phytohormone IBA. The second subgroup (S1.B) is composed of compounds **1**–**3**, which show a moderate growth-stimulating activity. 

The saponin with the best growth enhancement values was agameroside I (**4**) and this gave up to 38% coleoptile growth stimulation at 33 µM and maintained a certain level of stimulation up to 1 µM. The results for this compound are comparable to those obtained for the phytohormone IBA. Saponin **2** and cantalasaponin-1 (**3**) (Figure 1) are structurally related to agameroside I (**4**) but they have lower activation profiles, which indicates that the absence of glycosylation at C-3 in the spirostan-3,6-diols favors the stimulatory activity on wheat coleoptiles. 

Furthermore, compound **5** (which belongs to the same subgroup as IBA and compound **4**) displayed a good stimulation activity profile with a stimulation maximum at a concentration of 33 µM (29%) that is maintained up to 3.3 µM (16%).

### 2.2. Bio-Guided Fractionation of Yucca Schidigera

**Figure 5:**
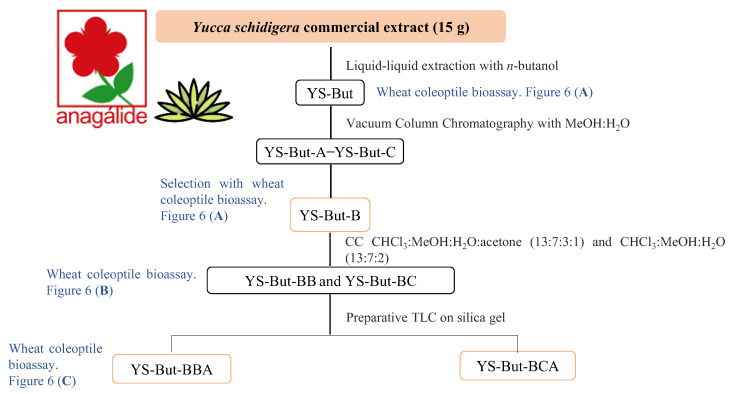
Bio-guided fractionation of the commercial extract of *Y. schidigera*.

**Figure 6:**
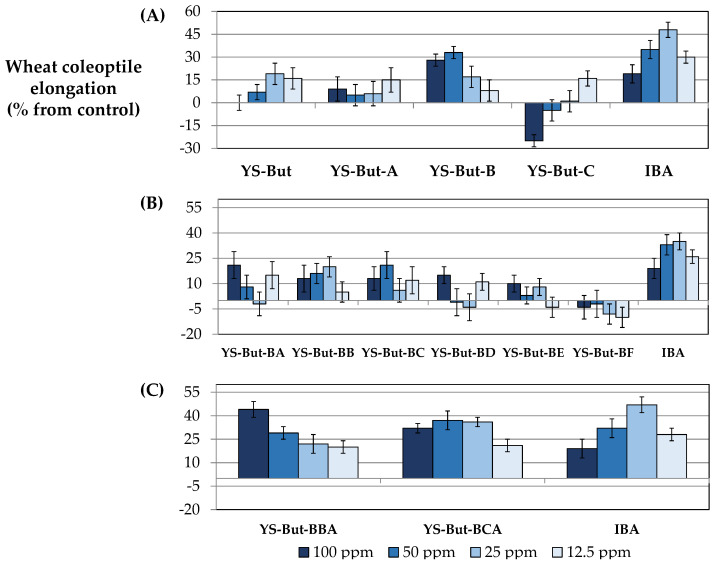
Wheat coleoptile bioassay for the first (**A**), second (**B**), and third (**C**) fractionations of *Y. schidigera* commercial extract using IBA as internal standard. Experiments were performed in triplicate and values are expressed as percentage difference from control and as mean ± SD. Coleoptile elongation was measured where negative values indicate growth inhibition and positive values represent growth stimulation.

Initially, a saponin crude extract (YS-But) from commercial *Yucca* extract was obtained by liquid–liquid extraction (water/*n*-butanol). This was evaluated in the wheat coleoptile bioassay (Figure 5 and Figure 6A) using the phytohormone indole-3-butyric acid (IBA) as positive control. The results indicate a moderate growth-stimulation activity. This first crude extract (YS-But) was subsequently partitioned by vacuum column chromatography using different ratios of H_2_O:MeOH as eluent. Three main fractions were obtained (YS-But-A–YS-But-C) and the bioactivity from this first separation step revealed that fraction YS-But-B showed the best growth-stimulating activity, with more than 30% stimulation achieved at 50 ppm and better activity values obtained when compared to the initial saponin crude extract YS-But (Figure 6A). Furthermore, a hormesis profile was observed for fraction YS-But-C, with inhibition at higher doses and 16 ± 5% stimulation achieved at lower concentrations. Therefore, this fraction was tested at lower doses (6.25 and 3.125 ppm), but in these cases significant effects were not observed (data not shown). 

The fraction YS-But-B was therefore selected for further purification by column chromatography on silica gel to give six fractions, which were evaluated again in the search for saponins with stimulatory activity. The results showed that the fractions YS-But-BB and YS-But-BC had the best growth-stimulating activity profiles (Figure 6B). These fractions were also obtained in the largest amounts and they were therefore selected to continue the bio-guided fractionation to yield two highly purified enriched mixtures (YS-But-BBA and YS-But-BCA). These two saponin-rich fractions showed good activity profiles, with stimulation values up to 40% obtained at the highest concentrations, and the activity values were similar to those of the positive control (IBA) (Figure 6C). It is worth mentioning, especially for fraction YS-But-BCA, that the stimulation levels were maintained even at the lowest concentrations.

### 2.3. Characterization of YS-But-BBA and YS-But-BCA Fractions

Analysis of the UPLC-MS chromatograms for these two fractions displayed molecular ions and fragmentations that were consistent with steroidal saponin features (Table 1). The fragmentation patterns usually observed for this kind of compound in negative mode are the precursor ion forming adduct with formate [M + HCOO]^–^ and [M–H]^–^, the different losses of sugar residues and, as the last fragment, the aglycone with the sugar moiety directly linked to it, which is commonly a hexose residue [Aglycone–H + 162]^–^ [23].

**Figure 7:**
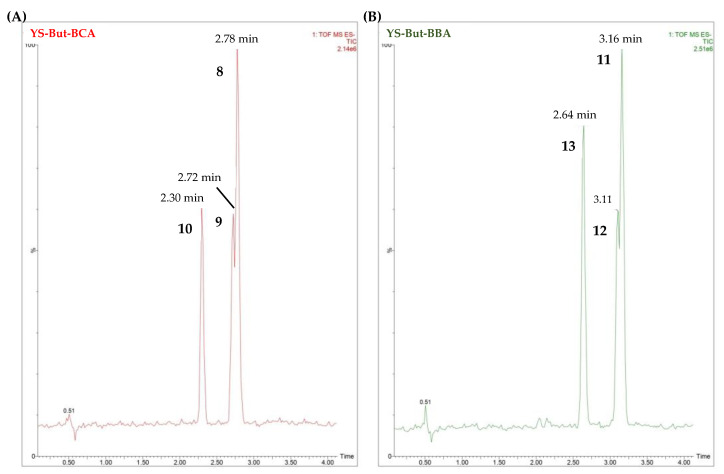
UPLC-MS chromatograms of fractions YS-But-BCA (**A**) and YS-But-BBA (**B**).

In both chromatograms (Figure 7), evidence for the three main compounds can be observed. Regarding the most active fraction (YS-But-BCA), compounds **8** and **9** gave the same molecular formula, which indicates that they are isomers, while compound **10** displayed a molecular formula without two hydrogens, thus suggesting the presence of an unsaturation. All three compounds showed fragmentation in agreement with three hexose residues. 

The ^1^H-NMR spectrum of the YS-But-BCA fraction displayed characteristic signals for steroidal saponins. Methyl groups signals (singlets for CH_3_-18 and -19, three were observed at 0.93 ppm and another three at 1.06 ppm, and doublets for CH_3_-21 and -27, at δ 0.67, 1.04, 1.31, and two at 1.35 ppm) in the downfield region corresponding to three aglycones were observed (Figure S1 in Supplementary Materials).

For aglycone identification, it is proposed to use the HMAI method (HMBC Method for Aglycone Identification) recently developed for the easy and reliable identification of the most representative aglycones in *Agave* species [24]. Most of structural features of *Yucca* aglycones are included in the identification method and HMAI has been successfully applied to saponin mixtures [17].

**Figure 8:**
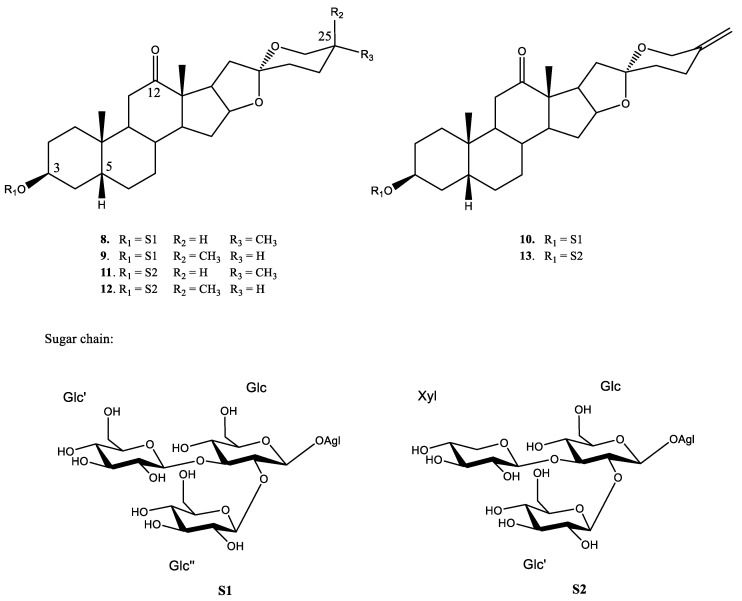
Structures of the spirostanic saponins elucidated from YS-But-BCA and YS-But-BBA fractions.

A comprehensive study of the HMBC correlations (Figure S3 in Supplementary Materials) and HMAI interpretation (Table 2, Figures S4 and S5, Tables S1 and S2 in Supplementary Materials) allowed the identification of signals that are consistent with the presence of a 25*R*, 25*S*, and 25(27)-dehydro-spirostanic-type aglycone with a carbonyl group at C-12 and H-5β. This information is consistent with the fragments *m*/*z* 591 and 589 ([Aglycone–H + 162]^–^) observed in the UPLC-MS analysis and it is deduced that fraction YS-But-BCA includes (25*R*)-3β-hydroxy-5β-spirostan-12-one (gloriogenin), (25*S*)-3β-hydroxy-5β-spirostan-12-one, and 3β-hydroxy-5β-spirost-25(27)-en-12-one (Figure 8).

In the downfield region of the ^1^H-NMR spectrum of fraction YS-But-BCA, only three anomeric signals at δ 4.84, 5.34, and 5.65 can be distinguished. This information showed the presence of three monosaccharides, which is consistent with the loss of three hexose units in the fragmentation pattern observed in the UPLC-QTOF/MS^E^ study of their three components (Figure 7A), and it is also congruent with the same sugar chain for all. The natures and connections of the different sugar units were elucidated by comparison of NMR data (Figures S6 and S7 in Supplementary Materials) [25] with those published for *Yucca schidigera* saponins [26]. Finally, the sugar chain bonded at C-3 of aglycones is β-d-glucopyranosyl-(1→2)-*O*-[β-d-glucopyranosyl-(1→3)]-*O*-β-d-glucopyranoside (S1-Figure 8).

On the basis of the results outlined above, it was established that the compounds with retention times of 2.78 min and 2.72 min are the isomers C-25*R* and *S* by comparison of the intensities of the C-27 signals in the ^1^H NMR spectra and the UPLC peak intensities. Finally, compound **8**, with a retention time of 2.78 min, is gloriogenin-3-*O*-{β-d-glucopyranosyl-(1→2)-*O*-[β-d-glucopyranosyl-(1→3)]-*O*-β-d-glucopyranoside} (YS-VII), the compound with a retention time of 2.72 min is (25*S*)-3β-hydroxy-5β-spirostan-12-one-3-*O*-{β-d-glucopyranosyl-(1→2)-*O*-[β-d-glucopyranosyl-(1→3)]-*O*-β-d-glucopyranoside} (**9**), and the compound with a retention time of 2.30 min is 3β-hydroxy-5β-spirost-25(27)-en-12-one-3-*O*-{β-d-glucopyranosyl-(1→2)-*O*-[β-d-glucopyranosyl-(1→3)]-*O*-β-d-glucopyranoside} (**10**). These compounds (Figure 8) have been previously described in *Y. schidigera* [26,27,28]. 

Regarding compounds **11**–**13**, the NMR data showed that these compounds have the same aglycone as saponins **8**–**10** and that the difference was in the sugar chain (Figure S2). Thus, the carbohydrate chain for these compounds differs from those described previously in the occurrence of a β-d-xylopyranose instead of a β-d-glucopyranose unit. These data are also consistent with the mass losses observed for fraction YS-But-BBA from the UPLC-QTOF/MS^E^ (the presence of two hexoses (162 Da) and one pentose residue (132 Da). Comparison of the results of NMR experiments (Figures S8 and S9 in Supplementary Materials) with published data for *Yucca schidigera* saponins [26] established the sugar chain as β-d-glucopyranosyl-(1→2)-*O*-[β-d-xylopyranosyl-(1→3)]-*O*-β-d-glucopyranoside (S2-Figure 8). Finally, the compound with a retention time of 3.16 min is gloriogenin-3-*O*-{β-d-glucopyranosyl-(1→2)-*O*-[β-d-xylopyranosyl-(1→3)]-*O*-β-d-glucopyranoside} (**11**), the compound with a retention time of 3.11 min is (25*S*)-3β-hydroxy-5β-spirostan-12-one-3-*O*-{β-d-glucopyranosyl-(1→2)-*O*-[β-d-xylopyranosyl-(1→3)]-*O*-β-d-glucopyranoside} (**12**) and, finally, the compound with a retention time of 2.64 min is 3β-hydroxy-5β-spirost-25(27)-en-12-one-3-*O*-{β-d-glucopyranosyl-(1→2)-*O*-[β-d-xylopyranosyl-(1→3)]-*O*-β-d-glucopyranoside} (**13**). These compounds (Figure 8) have also been reported previously in *Y. schidigera* [27,29,30]. 

### 2.4. Active Saponins in Yucca Schidigera Extract

**Figure 9:**
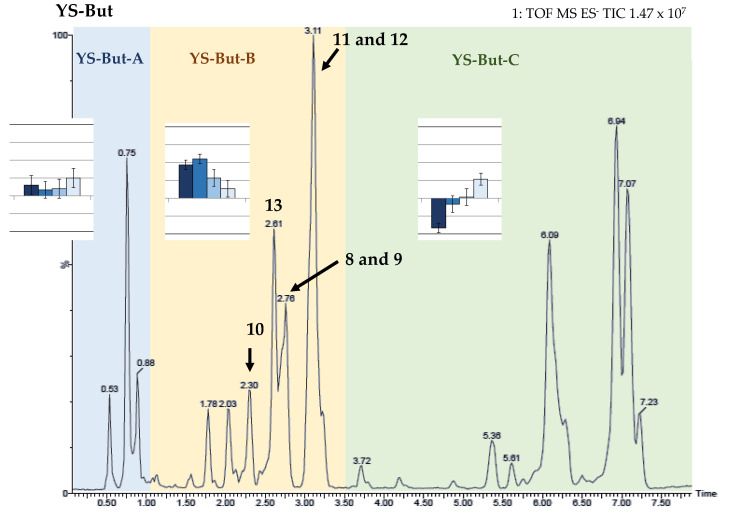
UPLC-MS chromatogram of the crude saponin extract YS-But. The chromatogram windows in which fractions YS-But-A to C and the active saponins were found are indicated.

Having characterized the saponins present in the two saponin fractions YS-But-BCA and YS-But-BBA, which showed the best growth stimulation activities, their relative abundance in the initial crude saponin extract (YS-But) was determined by UPLC-QTOF/MS^E^. This analysis allowed three main regions to be distinguished in the chromatogram and these are in accordance with the first fractions evaluated (YS-But-A–C) (Figure 9). Bio-guided fractionation led to the active saponins **8**–**13** and their structural elucidation. As can be observed in Figure 9, these saponins are found in the second region of the chromatogram.

In this way, the active saponins (**8**–**13**) represent 43% of the total of saponins in the crude extract YS-But (Table 3). Within this group of bioactive saponins, a 1:2.8 ratio was found between saponins with the sugar chains S1 (**8**–**10**) and S2 (**11**–**13**). 

The third region of the chromatogram also contains some major peaks at retention times from 5.0 to 7.0 min. The fragmentation patterns of these peaks are identical to those described for YS-But-BCA and YS-But-BBA fractions, with the exception that the fragments have a lower mass by 14 Da. This finding is in agreement with saponins with the same structures as **8**–**13** but without a carbonyl group in their aglycone backbone described for *Yucca schidigera* [27] (Table S3 in Supplementary Materials). Additionally, analysis of their relative abundance could demonstrate that, in this case, the saponins with S2 as the carbohydrate chain (compounds with retention times at 6.09, 6.94, and 7.07 min) had a higher abundance than those with S1 (5.36, 5.91, and 6.31 min). The ratio of these two groups was 10:1, respectively.

Fractions YS-But-BBA and -BCA showed good activity profiles (Figure 6C) with both types of sugar chain (S1 and S2, Figure 8). Nonetheless, a marked difference was observed between fractions YS-But-C and YS-But-B. The structural difference between the two fractions is a carbonyl group on C-12 of the aglycone skeleton. It can therefore be suggested that the presence of this oxygenation results in an increase in plant-growth stimulation.

## 3. Discussion

### 3.1. Stimulation Activity of Steroidal Saponins

Publications that describe the enhancement of plant growth usually concern compounds that are directly applied to plants or seedlings, where the effects are measured after several days and weeks. Stimulatory properties have also been evaluated for some products on etiolated wheat coleoptiles as a preliminary assay [31]. It is worth mentioning that the first studies performed with growth-regulating compounds were carried out on oat coleoptiles by Charles Darwin [32]. Subsequent studies were carried out on coleoptiles and it was postulated that there were substances that caused a phototropic response in the plant [33]. It was the plant physiologist Frits W. Went who finally showed that the apex of the coleoptile exerts its effect through a chemical substance and not through a physical inducement [34]. This substance was later identified as indole-3-acetic acid (IAA), a phytohormone present in most plants.

The etiolated wheat coleoptile bioassay (*Triticum aestivum* L.) was later optimized and used to evaluate the activity of auxins as compounds that regulate plant development. This bioassay was therefore chosen to evaluate the potential of steroidal saponins as growth promoters [20,21] using indole-butyric acid (IBA) as positive control [22].

The selection of steroidal saponins to be tested was based in the growth stimulation or hormesis [18,19] that have been observed on the roots of *L. sativa*. Saponins with a sugar chain of more than five units (**6** and **7**) showed the same hormesis profile on etiolated wheat coleoptiles that was previously observed in the phytotoxicity bioassay and is considered as an adaptive response that could be triggered by exposing plants to low levels of stressors such as herbicides through the stimulation of cellular defense mechanisms [35].

On the other hand, those saponins that showed the best root growth stimulation profiles on *L. sativa* (**2** and **4**) were selected [14,16] along with compound **3** due to its structural similarity to **2** and **4**. Moreover, since saponins with short sugar chains did not show inhibition profiles in phytotoxicity bioassays, saponin **5** was also included in this selection as it possesses a *cis* fusion of the rings A and B in the aglycone backbone (Figure 1).

These saponins showed a more defined stimulation activity profile when they were tested in the wheat coleoptile bioassay. Two saponins, **4** and **5**, showed interesting growth enhancement effects, with similar values obtained to the phytohormone IBA.

Brassinosteroids are a class of plant steroid hormones that modulate plant growth [36]. Their structural similarities with steroidal sapogenins such as diosgenin allowed the synthesis of brassinosteroid analogs [37,38]. Some of these derivatives have shown biological activity similar to natural brassinosteroids, thus highlighting their good results as growth-stimulating compounds.

**Figure 10:**
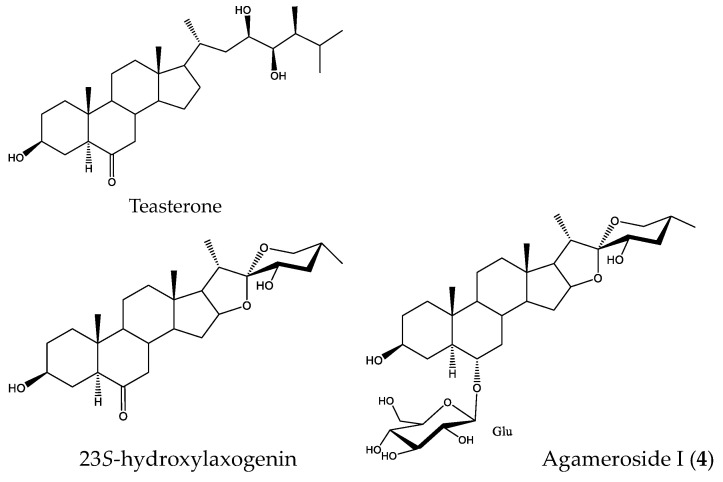
Structures of the brassinosteroid teasterone, its synthetic analog 23*S*-hydroxylaxogenin, and the saponin agameroside I (**4**).

The stimulatory activity of the sapogenin laxogenin, as well as its oxygenated synthetic derivatives at the C-23 position (23-ketolaxogenin and 23*S*-hydroxylaxogenin) (Figure 10), has been tested in vitro and in a field trial [39]. These compounds showed plant growth stimulation on *Raphanus sativus* (radish) seedlings. An increase in the length of hypocotyls compared to control was produced and this effect was greater for 23*S*-hydroxylaxogenin. Likewise, a strong increase in the germination of orange seeds was observed when they were treated with these compounds, and the most notable effects were also observed for the corresponding oxygenated analogs at C-23.

Agameroside I (**4**) has certain structural features similar to the steroid 23*S*-hydroxylaxogenin, which has been proposed as a synthetic analog of the brassinosteroid teasterone. As a consequence, the stimulatory activity of compound **4** could be related to some type of interaction with brassinosteroid metabolism. In fact, the influence of these phytohormones on optimal root growth has been described and this could be related to the effects observed on the roots of *L. sativa* [40].

**Figure 11:**
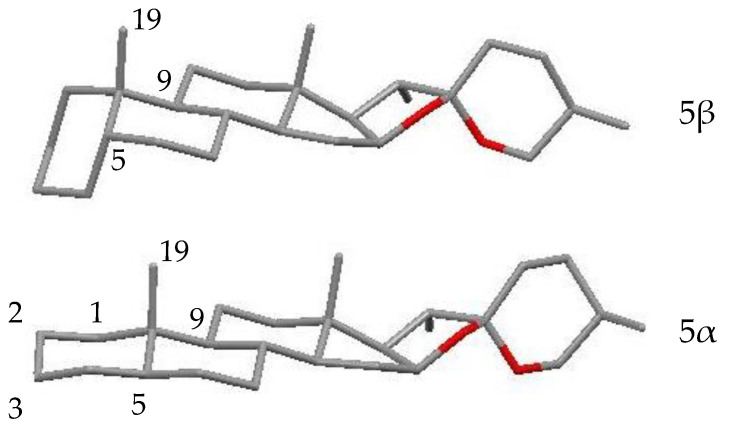
Carbon structure of aglycones with H-5β (*cis* fusion of A/B rings) and H-5α (*trans* fusion of A/B rings). The PCModel 9.2 application was used to represent the 3D models.

On the other hand, the chemical structure of compound **5** differs from that of **4** since it does not have oxygenation at C-6 and it is glycosylated at C-3. Moreover, when H-5 is β-oriented, a very different spatial arrangement of rings A and B is generated when compared with the rest of the tested saponins, which contain a *trans* fusion (H-5α) of rings A and B (Figure 11).

The plant growth-stimulating activity produced by some steroidal saponins is better than that exhibited by the triterpene saponin soyasaponin I (**1**). 

Of the two most active saponins, agameroside I (**4**) has not been described as the main product found in Agavaceae species. However, saponins with H-5β are the main components of most *Yucca* species [41] and the *Littaea* subgenus within the genus *Agave* [17,42]. To obtain steroidal saponins with the structural requirements to possess plant growth stimulation, *Y. schidigera* could be a promising source of these metabolites.

### 3.2. Yucca schidigera as a Commercial Source of Growth Stimulating Saponins

*Yucca schidigera* Roezl. belongs to the Agavaceae family and is native to the southwestern United States and northern Mexico. This species is one of the main sources of steroid saponins and their extracts are commercialized and approved by the Food and Drug Administration (FDA) as GRAS (Generally Recognized as Safe). These extracts are widely used as animal feed and cosmetics additives, as well as fertilizers to achieve rapid seed germination and root growth in agriculture [43]. 

The most abundant components identified in the commercial extracts of this species are monodesmosidic spirostanic saponins, as previously mentioned, and it is worth highlighting aglycones with H-5β that are usually linked to a di- or trisaccharide residue [27].

Due to the broad applications of *Yucca schidigera* extracts, it is worth mentioning their uses as fertilizers, ready availability, safety, and the structural characteristics of their saponins. Given these advantages, a bio-guided isolation of a commercial *Yucca* extract was proposed to obtain a saponin-enriched fraction with plant growth stimulation bioactivity. This allowed the elucidation of the structures of the bioactive saponins, which represent 43% of the total of saponins. These compounds have the structural requirements to show growth enhancement, as mentioned above, with an additional carbonyl group at C-12 on the aglycone backbone. Furthermore, these saponins show more marked growth-enhancement effects than those saponins without this functionalization. 

It is worth noting that the isolation and purification of this kind of compound remains a challenge [27]. For this reason, the application of saponin-enriched fractions with known chemical compositions on a large scale would be an alternative to the use of pure saponins. A knowledge of the structural characteristics of saponins required for growth stimulation could be applied as a quality control measure for commercial products of *Y. schidigera* extracts, which are used as fertilizers, by analyzing the presence of steroid saponins with a carbonyl group. In contrast, saponins of fraction YS-But-C showed good antifungal effects [41] and the *n*-butanol extract at 25 ppm could be used for two applications, namely, to improve crop yield and to achieve fungal control [44], because all saponins in this concentration had stimulatory activity (Figure 5A). On the other hand, a 200 ppm solution (0.2 g/L) of the commercial extract contains 25 ppm of the appropriate concentration of saponins (14%).

## 4. Materials and Methods

### 4.1. General Experimental Procedures

NMR spectra were recorded on an Agilent INOVA-600 spectrometer equipped with a 5 mm ^1^H–^13^C–^15^N cryoprobe. The ^1^H (599.772) and ^13^C (150.826) NMR spectra were recorded in pyridine-*d_5_* (Merck, Darmstadt, Germany) at room temperature. The chemical shifts are given on the δ scale and are referred to the residual pyridine (δ_H_ 8.70, 7.55, 7.18 and δ_C_ 149.84, 135.60, 123.48). For spectroscopic data, see Supplementary Materials (Figures S1–S3, S6–S9). Acetic acid and *n*-butanol were supplied by Panreac Química S.A. (Castellar del Vallés, Barcelona, Spain). Methanol and chloroform were obtained from VWR International (Radnor, PA, USA). TLC silica 60 F_254_ and TLC Si gel F_254_S RP-18 plates provided by the same commercial supplier were used to monitor the isolation processes. The compounds were visualized after spraying plates with H_2_SO_4_/H_2_O/HOAc (4:16:80 *v/v/v*). LiChroprep^®^ RP-18 (40–63 µm) from Merck (Darmstadt, Germany) was used for vacuum column chromatography for the first fractionation. For further purification, silica gel 0.060–0.200, 60 Å from Acros Organics (Geel, Belgium), and preparative TLC silica gel 60 F_254_ (0.25 mm) and TLC Si gel RP-18 F_254_S (0.25 mm) supplied by Merck (Darmstadt, Germany), were used.

### 4.2. Plant Material

*Y. schidigera* extract was supplied by Anagalide S.A. (CIF A22007728, Huesca, Spain) imported from Mexico with batch number Y-501816. Technical specifications were as follows: juice from the stem was pressed and processed in origin (Mexico) on 18 September 2018. Chemical composition: saponins (14%), 50°Bx and sodium sorbate (0.125%).

### 4.3. Extraction and Isolation

The commercial extract of *Y. schidigera* supplied by Anagalide S.A. (15.0 g) was dissolved in water (600 mL). A liquid–liquid extraction with *n*-butanol was performed in triplicate with 200 mL each time. The solvent was removed from the *n*-butanol layer under reduced pressure to yield 1.815 g (12.1%) of crude extract (YS-But). This residue was further purified by vacuum column chromatography with reverse phase silica gel (RP-18) with different ratios of H_2_O:MeOH (100% H_2_O, H_2_O:MeOH (4:6), H_2_O:MeOH (3:7), and 100% MeOH). Three main fractions were collected (YS-But-A–YS-But-C) and combined based on similar TLC patterns, and the fractions were assayed in the wheat coleoptile bioassay. The most active fraction (YS-But-B, 240.8 mg, 1.6% yield) was selected for further separation. YS-But-B was purified by column chromatography on silica gel using a biphasic solvent as eluent (CHCl_3_:MeOH:H_2_O:acetone and CHCl_3_:MeOH:H_2_O in a ratio of 13:7:3:1 and 13:7:2, respectively). The organic layer from this biphasic mixture was used as eluent. The fractions obtained in the largest amounts and with the best growth enhancement activity profiles (YS-But-BB, 35.1 mg, 0.2% yield, and YS-But-BC, 30.0 mg, 0.2% yield) were subjected to preparative TLC on silica gel 60 F_254_ 0.25 mm plates (using as the organic phase a mixture of CHCl_3_:MeOH:H_2_O (13:7:2) and ethyl acetate:AcOH:H_2_O (7:2:2) as eluents). This step led to two fractions named YS-But-BBA (12.4 mg, 0.083% yield) and YS-But-BCA (4.8 mg, 0.032% yield) and these were characterized by NMR and UPLC-MS analysis. 

### 4.4. UPLC-QToF/MS^E^ Analysis

The exact masses of the saponins were measured using the method previously described in the bibliography [17,23]. A UPLC-QTOF ESI (Waters Xevo G2, Manchester, UK) high-resolution mass spectrometer (HRESI-TOFMS) was used to measure the exact masses. The ultrahigh-performance liquid chromatograph was equipped with an ACQUITY UPLC^®^ HSS T3 1.8 µm, 2.1 × 100 mm column attached to an ACQUITY UPLC^®^ HSS T3 1.8 µm, 2.1 × 5 mm VanGuard precolumn maintained at 45 °C. The mobile phases (LC/MS grade) were water (A) and acetonitrile (B), each containing 0.1% (v/v) formic acid. The elution conditions were as follows: 60% A (0–0.5 min); A from 60% to 50% (0.5–6.0 min); A from 50% to 5% (6.0–7.0 min); 5% A (7.0–7.5 min); A from 5% to 60% (7.5–8.0 min) and maintained 60% A (8.0–10.0 min) to condition the column for the next injection. A constant 0.4 mL/min flow was applied. The autosampler temperature was set at 10 °C and the injection volume was 5 µL. 

Electrospray Ionization in the negative mode (ESI) was used with the following settings: sample probe capillary voltage 2800 V, cone voltage 30 V; source temperature 120 °C, and desolvation temperature 450 °C. Desolvation and cone gas with flow rates of 850 and 10 L/h were used, respectively. The data were acquired in the centroid mode using MS^E^ (low collision energy 6 eV, high collision energy ramp 20−80 eV) over a mass range of *m*/*z* 100−2000 and a retention time range of 0–10.0 min with 0.5 s scan time. The raw data files were processed using MassLynx version 4.1 (Waters Inc., Milford, MA, USA, 2013). The stock solutions (100 ppm) of the different saponin-rich fractions were prepared in water:methanol (6:4). All samples were filtered through a PTFE syringe filter (0.22 µm) prior to analysis.

### 4.5. Etiolated Wheat Coleoptile Bioassay

This assay was carried out according to the methodology previously described in the literature [45]. Wheat seeds (*Triticum aestivum* L. cv. Catervo) were sown in 15 cm diameter Petri dishes moistened with water and grown in the dark at 22 ± 1 °C for 4 days. The etiolated seedlings were selected for size uniformity under a green safelight. The selected coleoptiles were placed in a Van der Wij guillotine, the apical 2 mm was cut off and discarded, and the next 4 mm of the coleoptiles was used for bioassay.

Compounds and saponin fractions were dissolved in DMSO (0.5% v/v) and dilutions were prepared in a phosphate−citrate buffer solution containing 2% sucrose adjusted to 1 mM, 333 μM, 100 μM, 33 μM, 10 μM, 3.3 μM, 1 μM, 333 nM, 100 nM, 33 nM, and 10 nM in IBA assay, to 100 μM, 33 μM, 10 μM, 3.3 μM, and 1 μM in pure saponins assay, and to 100, 50, 25, and 12.5 ppm in *Yucca schidigera* bioguided fractionation. Control samples (buffered aqueous solutions dissolved also in DMSO (0.5% v/v) without any test compound) and the commercial auxin indole-3-butyric acid (IBA, Sigma Aldrich, St. Louis, MO, USA) were used as internal references under the same conditions as saponins and fractions. Five coleoptiles were placed in each test tube containing 2 mL of test or control solutions (three replicates per dilution). Test tubes were kept in a shaker in constant rotation (6 rpm) for 24 h at 22 °C in the dark. After this time, the coleoptiles were placed in a template and digitally photographed. Coleoptile elongation was measured by the digitalization of the images, and the data were analyzed statistically using Welch’s test.

### 4.6. Statistical Analysis

Complete linkage cluster analysis was used to group compounds with similar growth enhancement behaviors and to associate behavior with chemical structure. These analyses were performed based on squared Euclidean distances obtained by using Statistica v. 7.0 software (Tulsa, OK, USA). Coleoptile elongation expressed as percentage difference from control at all the concentrations tested and for all the assayed compounds was included in the cluster analysis. 

## 5. Conclusions

Steroidal saponins such as triterpenoid saponins can act to improve plant growth and development; nevertheless, the plant growth stimulation effects of steroidal saponins is rarely described in the literature. It has been noted that examples with a sugar chain of more than five units have a hormesis profile and only show stimulatory activity at low doses. Saponins that contain a sugar chain with fewer sugar residues show a more defined stimulation activity profile and *Y. schidigera* extract constitutes an attractive source for these metabolites. Furthermore, it has been demonstrated that the wheat coleoptile bioassay is an appropriate method to measure plant growth stimulation, since this is a reliable, rapid, and highly sensitive process to measure this type of bioactivity.

Bio-guided fractionation of *Y. schidigera* extract led to the obtention of two saponin-rich fractions of defined composition with good stimulation activity profiles.

Commercial extracts or enriched saponins fractions of *Y. schidigera* [27,41] could be used to improve crop yield [44] without the need to isolate the pure compound, since the isolation and purification of this kind of compounds is not sustainable and economically viable.

## Figures and Tables

**Table 1:** UPLC-QTOF/MS^E^ data for YS-But-BCA and YS-But-BBA fractions.

Saponin-Enriched Fraction	Compounds	Retention Time (min.)	[M–H]^–^	Fragmentation (*m*/*z*)	Molecular Formula [M–H]^–^	mDa
YS-But-BCA	**8**	2.78	915.4567	753.4037, 591.3527	C_45_H_71_O_19_	−0.6
	**9**	2.72	915.4586	753.4008, 591.3475	C_45_H_71_O_19_	−2.3
	**10**	2.30	913.4427	751.3886, 589.3322	C_45_H_69_O_19_	−0.6
YS-But-BBA	**11**	3.16	885.4481	753.4009, 591.3557	C_44_H_69_O_18_	−0.8
	**12**	3.11	885.4537	723.3862, 591.3455	C_44_H_69_O_18_	−0.3
	**13**	2.64	883.4299	751.3875, 589.3236	C_44_H_67_O_18_	−2.8

**Table 2:** Correlations between methyl groups and nearby carbons observed in the HMBC spectra for fractions YS-But-BCA and YS-But-BBA, as well as HMAI interpretation.

^1^ H NMR Signal	HMBC Correlations			Methyl Assig.	Flowchart Information
4.77 and 4.80 (D14)					SP C25 DB
1.35 d	42.9	54.4 (D6)	109.5 (D5)	C-21	SP C12 CO
1.31 d	42.6	54.4 (D6)	109.5 (D5)	C-21	SP C12 CO
1.04 d (D2)	26.2 (D1)	27.6	65.2 (D1)	C-27	SP C25 *S*
0.67 d (D2)	29.1 (D1)	30.6	66.9 (D1)	C-27	SP C25 *R*
1.06 s (S2)	54.2	55.8	213.1 (S1)	C-18	SP C12 CO
0.93 s	30.4 (S10)	36.0	41.9 (S9)	C-19	H-5β

^1^ C#: position with functionalization; SP: spirostanic; β, *R*, *S*: configurations; DB: double bond; CO: carbonyl group; S#: decisions in singlets flowchart; D#: decisions in doublets flowchart.

**Table 3:** Relative abundances of compounds **8**–**13** in the saponin crude extract YS-But.

Sugar Chain	Compounds	Retention Time (min)	[M + HCOO]^–^	Relative Abundance ^1^
S1	**8/9**	2.76	961.4646/961.4651	8.5
	**10**	2.30	959.4451	3.0
S2	**11/12**	3.11	931.4529/931.4554	23.4
	**13**	2.61	929.4365	8.9

^1^ Percent area of trace vs. total area of saponin peaks.

## Supplementary Materials

The following supporting information can be downloaded at: https://www.mdpi.com/article/10.3390/plants11233378/s1, Table S1: HMAI Table of ^13^C NMR chemical shifts for doublets. Table S2: HMAI Table of ^13^C NMR chemical shifts for singlets. Table S3: UPLC-QTOF/MS^E^ data for the saponins in the chromatogram window from 5 to 8 min of the crude extract YS-But. Figure S1: ^1^H NMR spectrum of fraction YS-But-BCA. Figure S2: ^1^H NMR spectrum of fraction YS-But-BBA. Figure S3: HMBC spectrum of fraction YS-But-BCA and YS-But-BBA. Figure S4: HMAI Flowchart of doublets with notes. Figure S5: HMAI Flowchart of singlets with notes. Figure S6: TOCSY-1D spectra obtained selected anomeric protons of sugar chain S1. Figure S7: ROESY-1D spectra obtained selected anomeric protons of sugar chain S1. Figure S8: TOCSY-1D spectra obtained selected anomeric protons of sugar chain S2. Figure S9: ROESY-1D spectra obtained selected anomeric protons of sugar chain S2.
