# Supplementary material for: Steroidal Saponins with Plant Growth Stimulation Effects; Yucca schidigera as a Commercial Source
Source: Plants (Basel). 2022 Dec 5;11(23):3378. doi: 10.3390/plants11233378 (PMC9740418; doi:10.3390/plants11233378)
Supplement: Supplementary file 1 [file plants-11-03378-s001.zip › plants-2026689-supplementary.pdf]

Supplementary Materials

Steroidal saponins with growth stimulation effects. *Yucca schidigera* as a commercial source

Alexandra G. Durán[1], Juan M. Calle[1], Andy J. Pérez[2], Francisco A. Macías[1], Ana M. Simonet[1,]*

[1]Allelopathy Group, Department of Organic Chemistry, Institute of Biomolecules (INBIO), Campus de Excelencia Internacional (ceiA3), School of Science, University of Cadiz, C/ República Saharaui, 7, 11510 Puerto Real, Cadiz, Spain.

[2]Departamento de Análisis Instrumental, Facultad de Farmacia, Universidad de Concepción, Concepción, Chile.

* E-mail: ana.simonet@uca.es

Table of Contents

Table S1. HMAI Table of ^{13}C NMR chemical shifts for doublets 3

Table S2. HMAI Table of ^{13}C NMR chemical shifts for singlets 4

Table S3. UPLC-QTOF/MS^E data for the saponins in the chromatogram window from 5 to 8 minutes of the crude extract YS-But 5

Figure S1. ^1H NMR spectrum of fraction YS-But-BCA (600 MHz, Pyridine-*d5*) 6

Figure S2. ^1H NMR spectrum of fraction YS-But-BBA (600 MHz, Pyridine-*d5*) 7

Figure S3. HMBC spectrum for fractions YS-But-BCA and YS-But-BBA 8

Figure S4. HMAI Flowchart of doublets with notes 9

Figure S5. HMAI Flowchart of singlets with notes 10

Figure S6. TOCSY-1D spectra obtained selected anomeric protons of sugar chain S1 11

Figure S7. ROESY-1D spectra obtained selected anomeric protons of sugar chain S1 12

Figure S8. TOCSY-1D spectra obtained selected anomeric protons of sugar chain S2 13

Figure S9. ROESY-1D spectra obtained selected anomeric protons of sugar chain S2 14

Table S1. HMAI Table of ^{13}C NMR chemical shifts for doublets. HMBC correlations with doublet signals of methyl groups C-21 and C-27. (From Simonet et al., *Phytochem. Anal.*, 2021: *32*, 38-61).

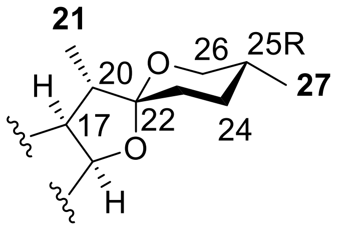

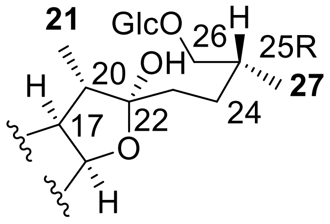

Structural Features						HMBC signals								
C-9	C-12	C-23	C-24	C-22	C-25	**H-27**	C-24	C-25	C-26	**H-21**	C-17	C-20	C-22	Data
				SP	R	0.67	29.1	30.6	66.7	1.12	62.9	41.9	109.3	**1**
				SP	DB	-	-	-	-	1.08	63.2	41.9	109.4	**11**
				F	R	0.96	28.5	34.4	75.3	1.30	63.9	40.8	110.7	**43**
				FM	R	0.98	28.2	34.2	75.2	1.16	64.1	40.5	112.7	**44**
		OHα		SP	R	0.72	38.9	31.8	66.0	1.16	62.6	35.9	111.7	**58**
			OGlc*β*	SP	R*	1.12	81.5	38.2	65.1	1.02	62.3	42.1	111.6	**62**
		OHα	OGlc*β*	SP	R*	1.19	87.9	37.9	64.1	1.15	62.0	34.6	112.7	**60**
	CO			SP	S	1.05	26.2	27.5	65.2	1.35	54.2	43.1	109.8	**24**
	CO			SP	R	0.64	29.2	30.5	66.9	1.31	54.3	42.6	109.3	**18**
	CO			F	R	0.96	28.4	34.3	75.3	1.53	54.9	41.3	110.9	**32**
	CO			F	S	1.01	28.3	34.4	75.3	1.51	54.8	41.3	110.8	**34**
DB	CO			SP	R	0.67	29.2	30.5	67.0	1.38	54.5	43.0	109.5	**28**
	OH*β*			SP	R	0.67	29.4	30.7	66.9	1.41	63.0	43.1	109.6	**47**

OH: hydroxyl; DB: double bond; CO: carbonyl; SP: spirostanic; F: furostanic; R/S/*α*/*β*: chiral center configuration.

* R is the relative configuration; S is the absolute configuration because a glucopyranosyloxy moiety is at C-24.

Table S2. HMAI Table of ^{13}C NMR chemical shifts for singlets. HMBC correlations with singlet signals of methyl groups C-18 and C-19. (From Simonet et al., *Phytochem. Anal.*, 2021: *32*, 38-61).

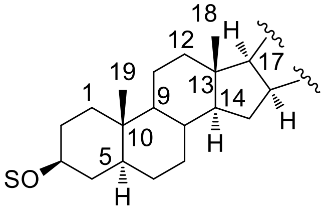

Structural Features								HMBC signals										
C-2	C-5	C-6	C-9	C-12	C-22	C-23	C-24	**H-18**	C-12	C-13	C-14	C-17	**H-19**	C-1	C-5	C-9	C-10	Data
	α				SP			0.80	40.0	40.5	56.1	62.9	0.62	36.9	44.4	54.1	35.6	**1**
	α				SP	OH*α*	OGlc*β*	1.01	40.7	41.4	56.6	62	0.75	37.5	45.6	54.6	35.9	**60**
	α			CO	SP			1.03	212.8	55.4	55.9	54.3	0.61	36.6	44.4	55.5	36.3	**18**
	α		DB	CO	SP			0.98	204.3	51.3	52.7	54.5	0.79	35.0	42.5	171.3	39.5	**28**
OH *α*	α		DB	CO	SP			0.97	204.3	51.4	52.7	54.6	0.86	43.5	42.5	170.5	40.6	**55**
	α			OH*β*	SP			1.06	79.3	46.6	55.2	63.0	0.64	37.2	44.7	53.6	35.9	**47**
OH *α*	α				SP			0.78	40.0	40.6	56.3	63.0	0.69	45.6	44.6	54.3	36.8	**35**
	β				SP			0.79	40.3	40.9	56.5	63.1	0.84	30.8	36.9	40.2	35.2	**4**
OH *β*	β				SP			0.77	40.2	40.8	56.3	63.1	0.87	40.5	36.4	41.4	36.9	**38**
	DB				SP			0.80	39.9	40.5	56.7	62.9	0.85	37.5	141.1	50.3	37.1	**9**
	DB			CO	F			1.13	212.9	55.4	56.0	54.9	0.91	37.0	140.9	52.4	37.6	**32**
OH *α*	DB				SP			0.78	39.8	40.5	56.5	62.9	0.91	45.8	140.1	50.2	38.0	**40**
OH *α*	DB				SP		OGlc*β*	0.71	39.7	40.4	56.5	62.3	0.91	45.7	140.1	50.1	37.9	**62**
OH *α*	DB				F			0.85	39.9	40.8	56.5	63.9	0.92	45.8	140.1	50.3	38.0	**43**
OH *α*	DB				FM			0.77	39.6	40.8	56.4	64.1	0.91	45.7	140.1	50.2	37.9	**44**
	α	OH*α*			SP			0.81	40.0	40.7	56.2	62.9	0.68	37.6	52.1	54.0	36.4	**45**
	α	OGlc*α*			SP			0.76	40.0	40.8	56.4	63.0	0.68	37.5	50.9	53.8	36.7	**46**
	α	OGlc*α*			SP	OH*α*		0.96	22.7	41.4	56.5	62.6	0.74	37.8	51.3	54.0	36.8	**58**

OH: hydroxyl; DB: double bond; CO: carbonyl; SP: spirostanic; F: furostanic; *α*/*β*: chiral center configuration.

Table S3. UPLC-QTOF/MS^E data for the saponins in the chromatogram window from 5 to 8 minutes of the crude extract YS-But.

**Retention time (min.)**	**$[M – H]^−$**	**Fragmentation (*m/z*)**
6.31	901.4745	739.4255, 577.3707
5.61	901.4756	739.4235, 577.3746
5.36	899.4613	737.4082, 575.3576
7.07	871.4670	739.4254, 577.3730
6.94	871.4661	739.4251, 577.3732
6.09	869.4522	737.4103, 577.3730

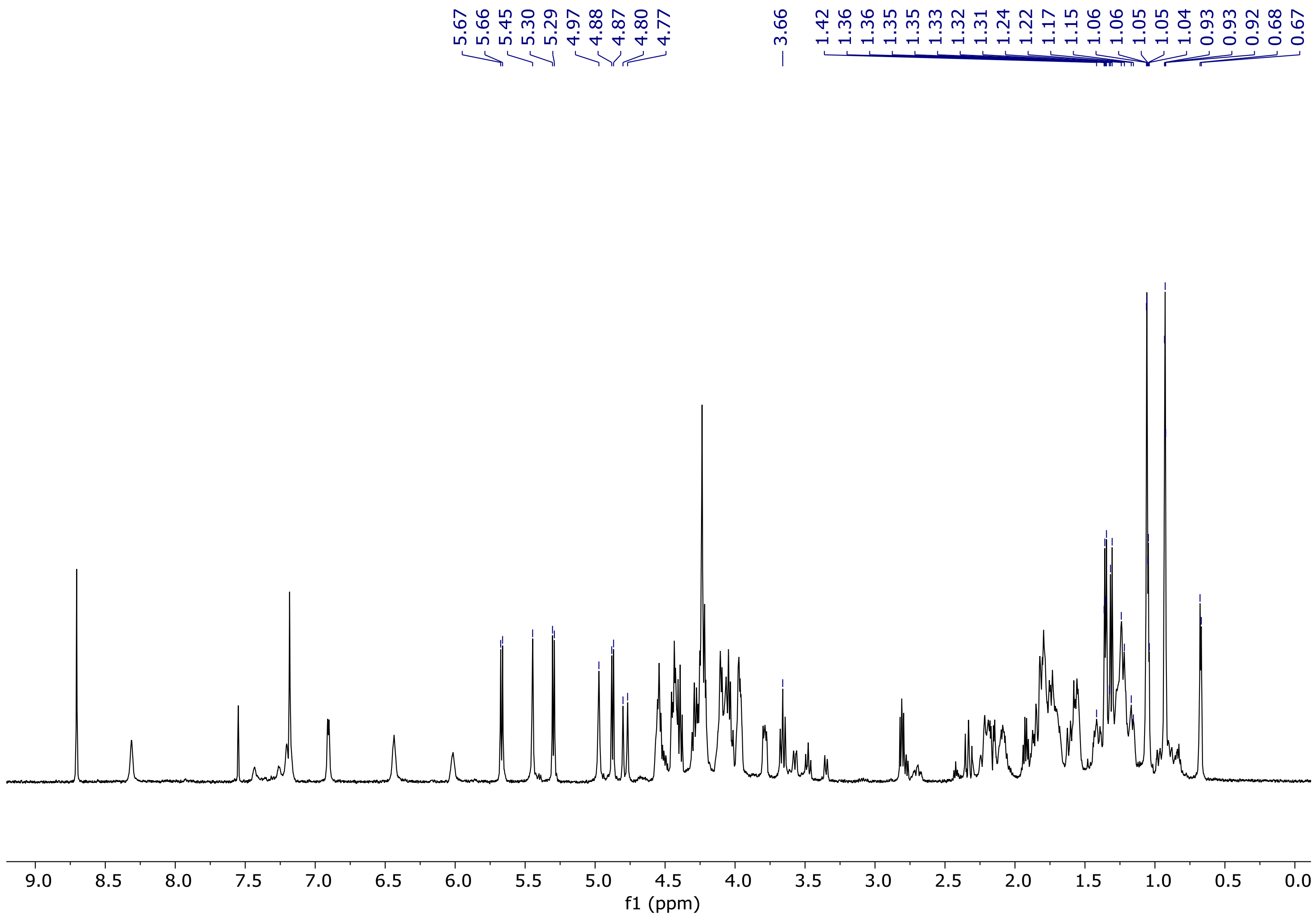

Figure S1. ^{1}H NMR spectrum of fraction YS-But-BBA (600 MHz, Pyridine-*d5*).

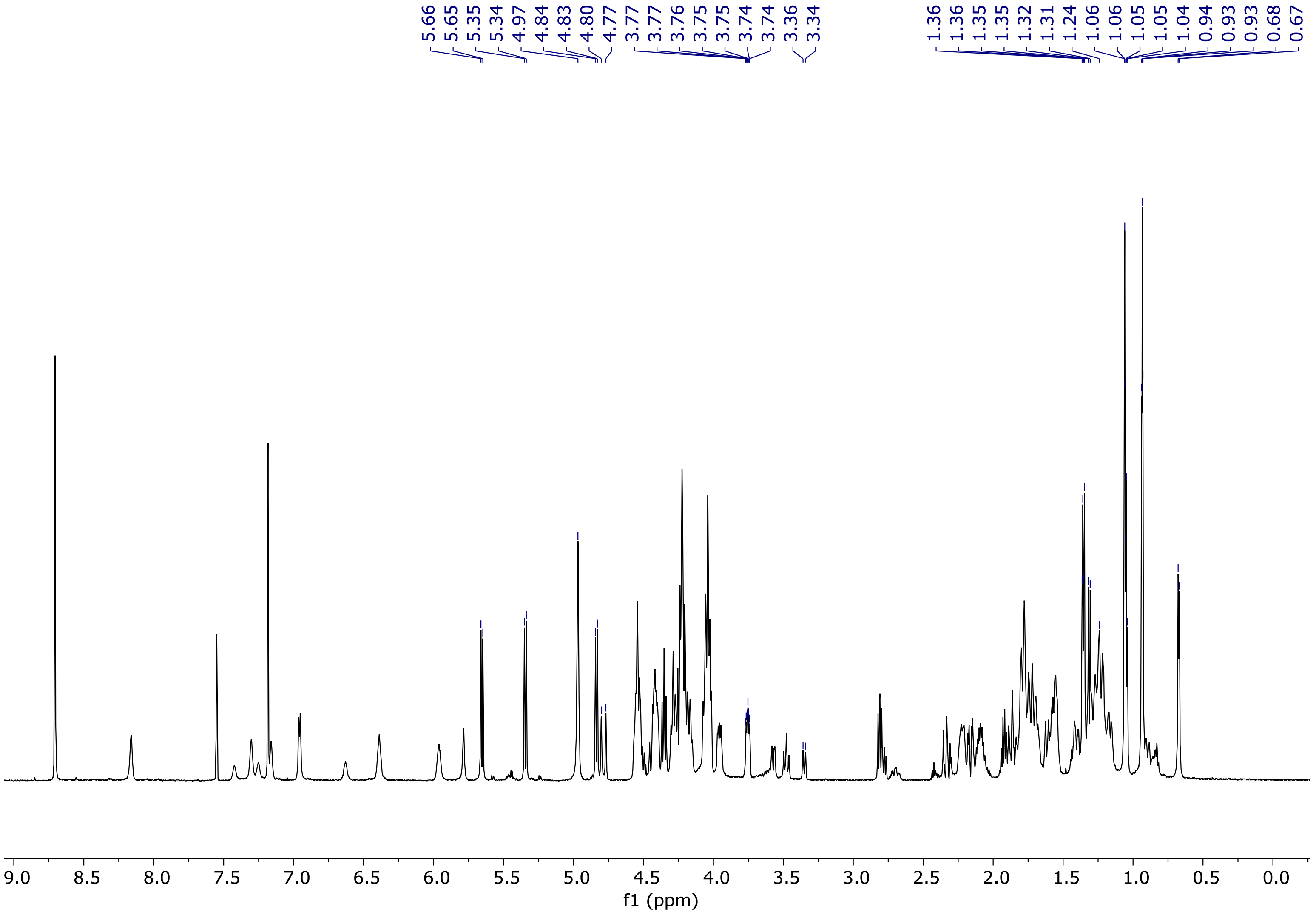

Figure S2. ^{1}H NMR spectrum of fraction YS-But-BCA (600 MHz, Pyridine-*d5*).

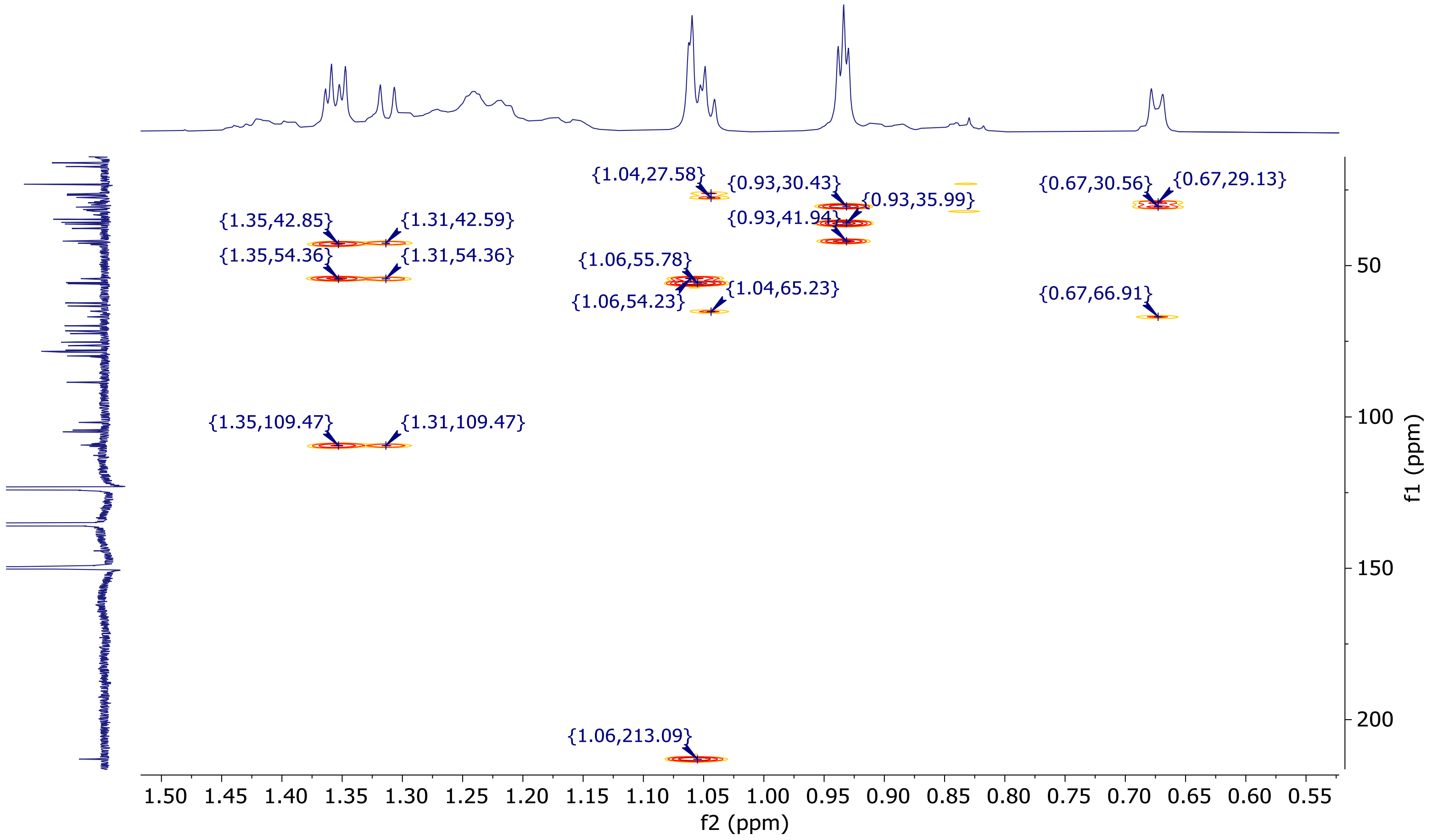

Figure S3. HMBC spectrum for fractions YS-But-BCA and YS-But-BBA (0.45 to 1.45 ppm; 600 MHz, Pyridine-*d5*).

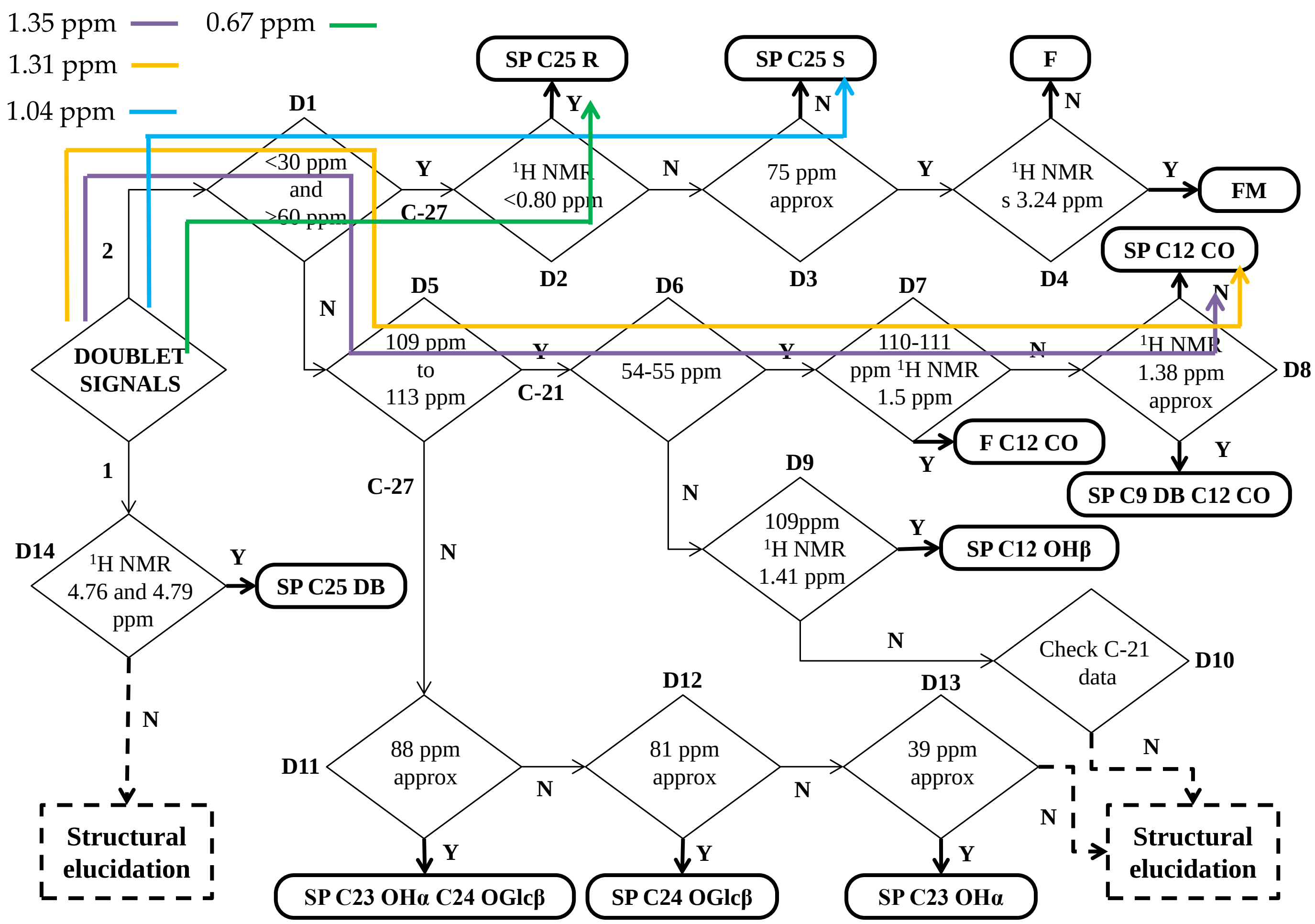

SP: spirostanic; F furostanic; FM: methoxyfurostanic; R/S/α/β quiral configuration; CO: carbonyl group; OH: hydroxyl group; DB: double bound; Oglc: glucopyranosyloxy; Y: Yes; N: Not.

Figure S4. HMAI Flowchart of doublets with notes for fractions YS-But-BCA and YS-But-BBA.

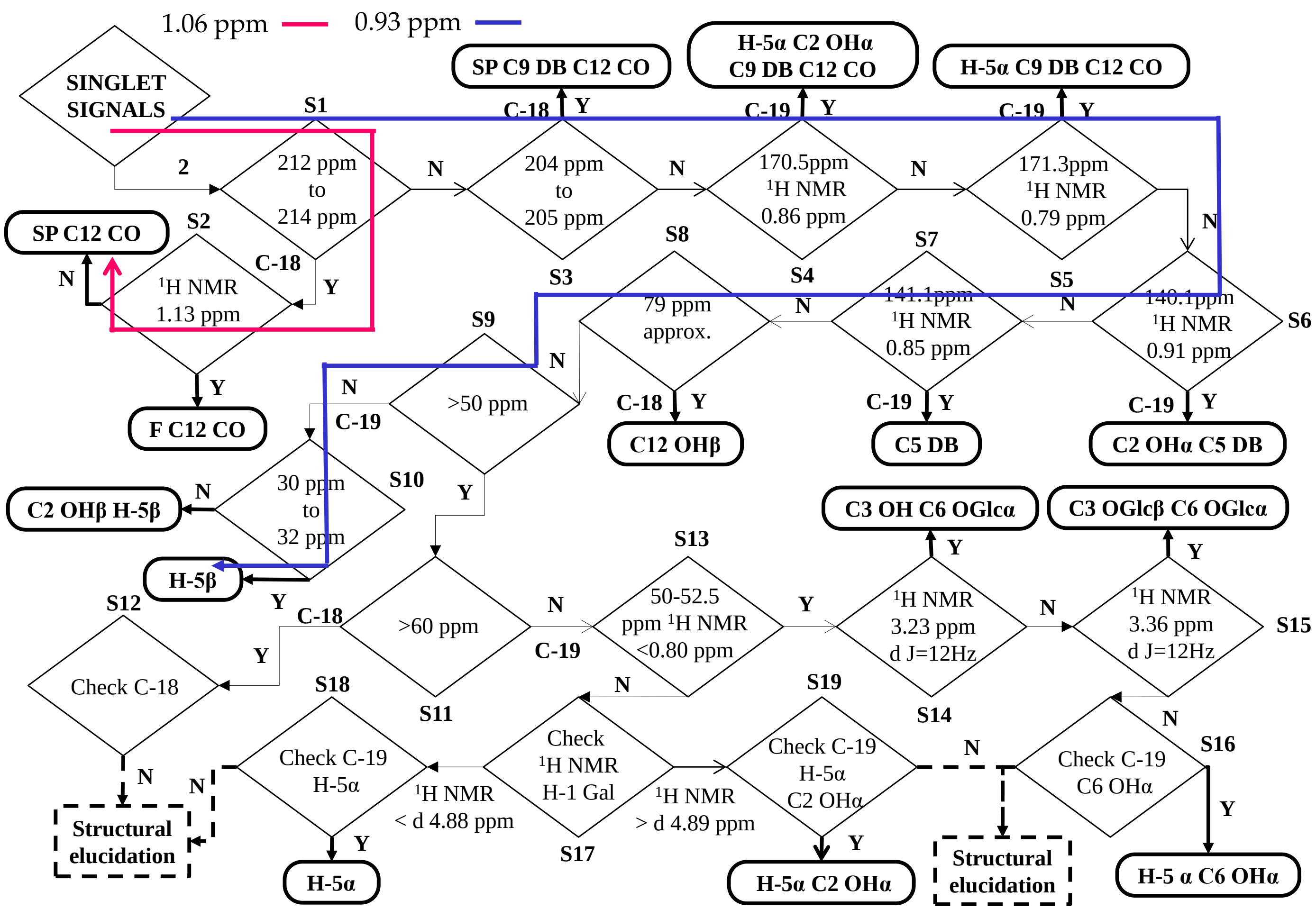

SP: spirostanic; F furostanic; R/S/α/β quiral configuration; CO: carbonyl group; OH: hydroxyl group; DB: double bound; Oglc: glucopyranosyloxy; Y: Yes; N: Not.

Figure S5. HMAI Flowchart of singlets with notes for fractions YS-But-BCA and YS-But-BBA.

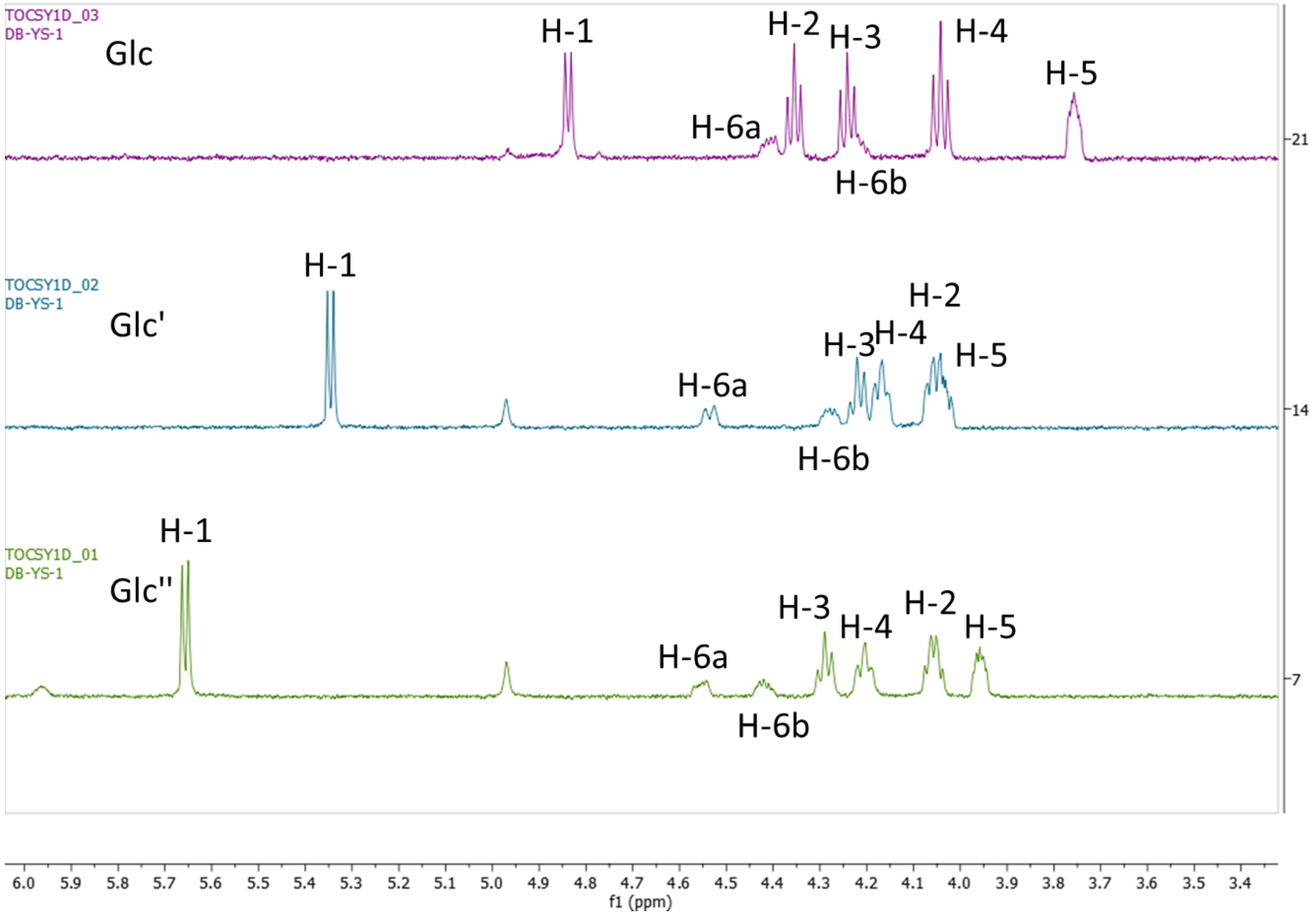

Figure S6. TOCSY-1D spectra obtained selected anomeric protons of sugar chain S1

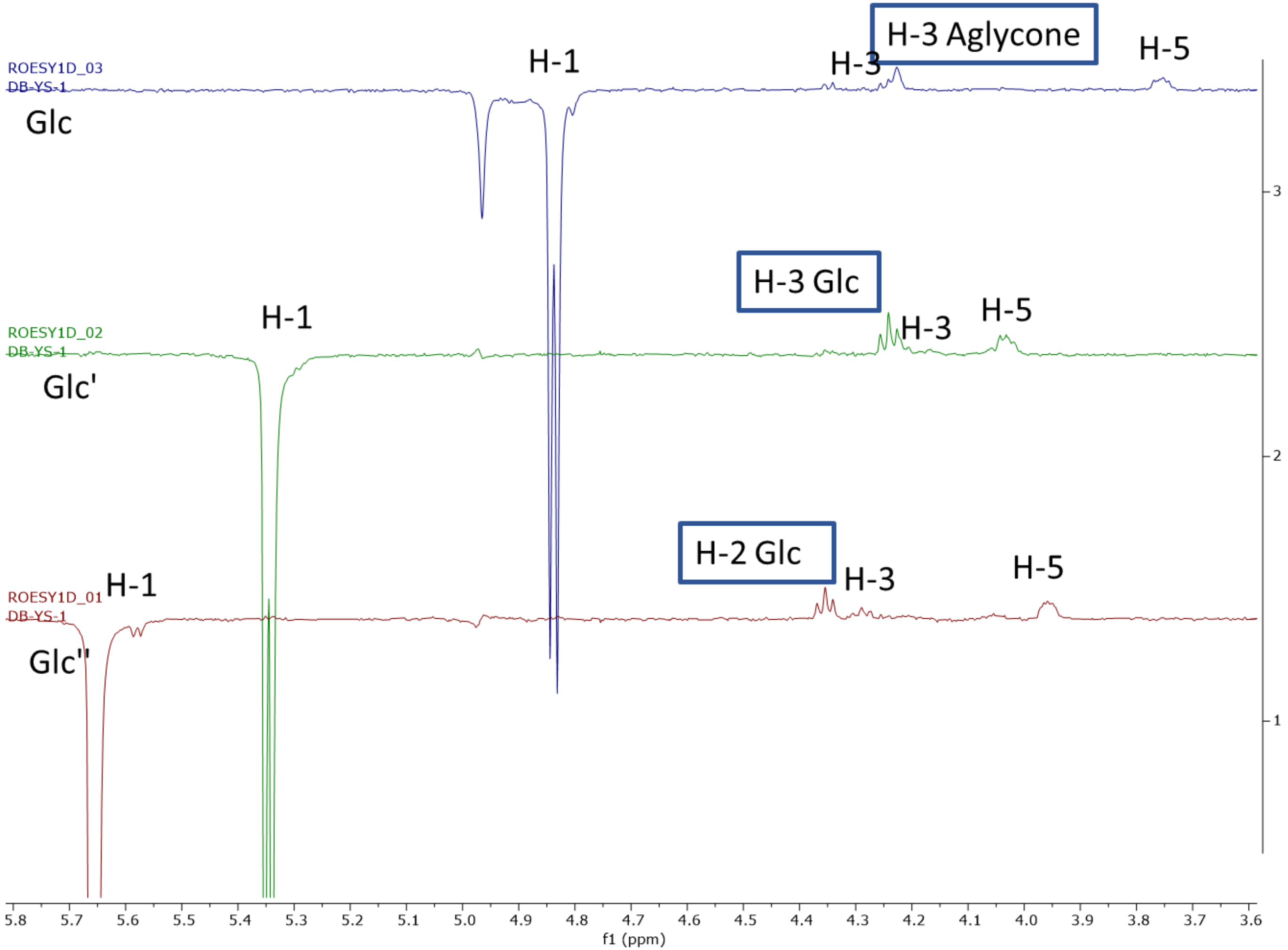

Figure S7. ROESY-1D spectra obtained selected anomeric protons of sugar chain S1

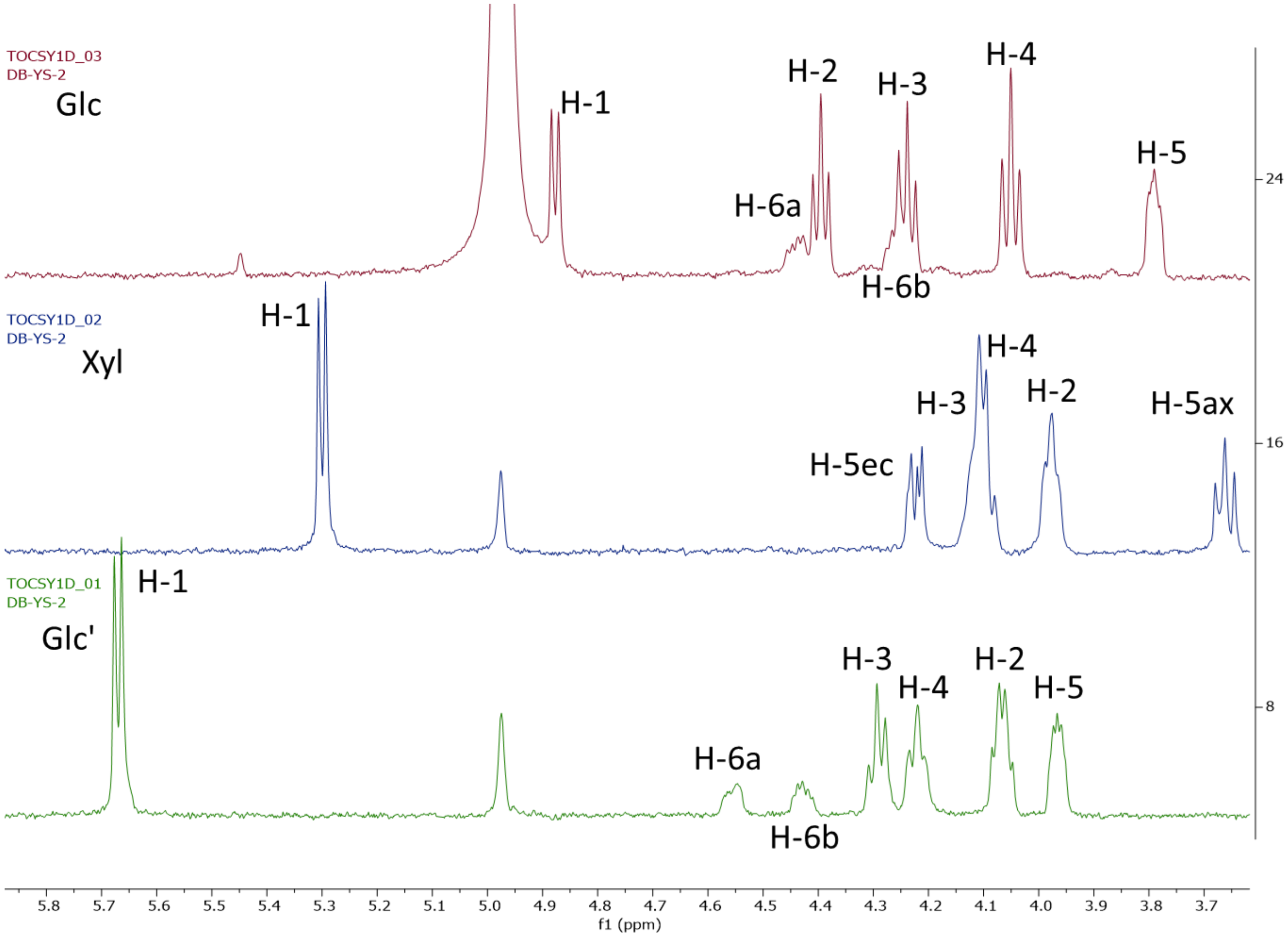

Figure S8. TOCSY-1D spectra obtained selected anomeric protons of sugar chain S2

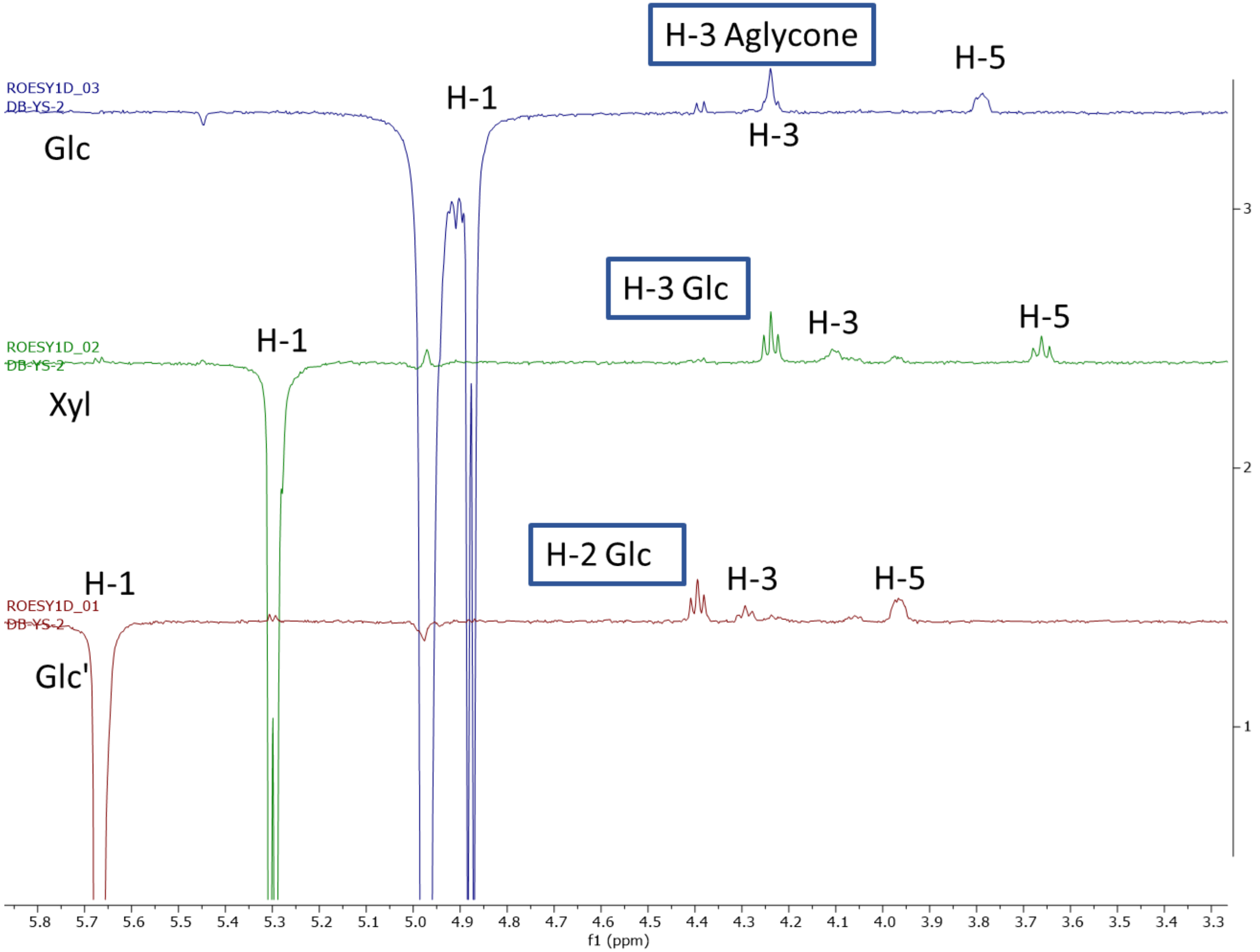

Figure S9. ROESY-1D spectra obtained selected anomeric protons of sugar chain S2
